# 免疫检查点抑制剂相关感染诊治建议

**DOI:** 10.3779/j.issn.1009-3419.2019.10.11

**Published:** 2019-10-20

**Authors:** 旻雅 陆, 丽 张, 玥 李, 汉萍 王, 潇潇 郭, 佳鑫 周, 炼 段, 晓燕 斯, 英春 徐, 力 张

**Affiliations:** 1 100730 北京，中国医学科学院，北京协和医院检验科 Department of Clinical Laboratory, Peking Union Medical College Hospital, Chinese Academy of Medical Sciences, Beijing 100730, China; 2 100730 北京，侵袭性真菌病机制研究与精准诊断北京市重点实验室 Beijing Key Laboratory for Mechanisms Research and Precision Diagnosis of Invasive Fungal Diseases (BZ0447), Beijing 100730, China; 3 100730 北京，中国医学科学院，北京协和医院消化内科 Department of Gastroenterology, Peking Union Medical College Hospital, Chinese Academy of Medical Sciences, Beijing 100730, China; 4 100730 北京，中国医学科学院，北京协和医院呼吸内科 Department of Respirology, Peking Union Medical College Hospital, Chinese Academy of Medical Sciences, Beijing 100730, China; 5 100730 北京，中国医学科学院，北京协和医院心内科 Department of Cardiology, Peking Union Medical College Hospital, Chinese Academy of Medical Sciences, Beijing 100730, China; 6 100730 北京，中国医学科学院，北京协和医院风湿免疫科 Department of Rheumatology and Immunology, Peking Union Medical College Hospital, Chinese Academy of Medical Sciences, Beijing 100730, China; 7 100730 北京，中国医学科学院，北京协和医院内分泌科 Department of Endocrinology, Peking Union Medical College Hospital, Chinese Academy of Medical Sciences, Beijing 100730, China

**Keywords:** 免疫检查点, PD-1/PD-L1抑制剂, 免疫治疗相关不良事件, 感染, Immune checkpoint, PD-1/PD-L1 inhibitors, Immune-related adverse events, Infections

## Abstract

免疫检查点抑制剂（immune checkpoint inhibitors, ICIs）已成功应用于多种恶性肿瘤治疗，其中程序化细胞死亡蛋白（programmed death 1, PD-1）/程序化细胞死亡配体-1（programmed death ligand 1, PD-L1）抑制剂近年来开始用于非小细胞肺癌中。目前认为PD-1/PD-L1抑制剂具有较小的副作用，并不会增加感染风险。但目前出现了免疫治疗相关不良事件（immune-related adverse events, irAEs）使用糖皮质激素和免疫抑制剂后出现机会性感染，及未出现irAEs的情况下发生潜伏/慢性感染重新激活的病案报道，使得免疫检查点抑制剂相关感染开始受到重视。本文将针对PD-1/PD-L1抑制剂相关感染的现有临床数据、可能机制及诊治建议进行叙述。

## Abstract (en)

Immune checkpoint inhibitors (ICIs) have been widely used in management of malignant tumor. Programmed death ligand 1 (PD-1)/programmed death ligand 1 (PD-L1) inhibitors have been introduced to treat non-small cell lung cancer (NSCLC) in recent years. Currently, PD-1/PD-L1 inhibitors are considered to have minor side effects and do not independently increase the risk of infection. However, they may cause immune-related adverse events (irAEs) that can require immunosuppressive therapy with corticosteroids and/or immunosuppressants, leading to opportunistic infections. Furthermore, there were reports about reactivation of chronic/latent infections without irAEs. Thus, immune checkpoint inhibitor related infections have drawn more and more attention in the world. In this paper, we described the potential mechanism, available clinical data and recommendation of diagnosis and management for PD-1/PD-L1 inhibitor related infections.

## 1 概述

程序化细胞死亡蛋白(programmed death 1, PD-1) /程序化细胞死亡配体-1 (programmed death ligand 1, PD-L1)抑制剂近年来开始用于非小细胞肺癌(non-small cell lung cancer, NSCLC)的免疫治疗。单独应用免疫治疗，或联合化疗进行治疗，目前已被列入进展期/晚期无表皮生长因子受体(epidermal growth factor receptor, *EGFR*）、间变性淋巴瘤激酶(anaplastic lymphoma kinase, *ALK*）或*ROS1*突变NSCLC的初始治疗中^[1]^。

PD-1/PD-L1抑制剂通常被认为具有较小的副作用。由于其促进T细胞效应体的功能，目前认为PD-1/PD-L1抑制剂的使用并不会增加感染的风险。然而，PD-1/PD-L1抑制剂治疗过程中，也出现了因免疫治疗相关不良事件(immune-related adverse events, irAEs)使用糖皮质激素等药物而产生暂时性免疫抑制所致机会性感染的病例报道^[2, 3]^。同时，目前也有报道PD-1/PD-L1抑制剂使用过程中未出现irAEs的情况下发生潜伏/慢性感染重新激活的病例^[4]^。

## 2 PD-1/PD-L1靶向药物的作用机制、适应证

PD-1是一个关键的免疫检查点，可抑制外周组织中T细胞的活性^[5]^。它主要于激活的CD4^+^、CD8^+^T细胞中表达，但也在B细胞、单核细胞、自然杀伤(natural killer, NK)细胞和树突细胞中表达^[6]^。PD-1可由PD-L1和PD-L2两种配体触发。任一配体与PD-1的结合均可强烈抑制CD8^+^T细胞效应区的功能。PD-L1可在肿瘤细胞表面和肿瘤微环境中存在的各种细胞表面表达。浸润肿瘤组织的T细胞分泌干扰素(interferon, IFN) -γ，触发了包括PD-L1表达在内的调节性免疫抑制循环，从而反馈性地使PD-1表达上调。阻断PD-1/PD-L1可以切断这种负循环，恢复抗肿瘤免疫^[7]^。PD-1/PD-L1表达强度被证实与多种肿瘤类型（如NSCLC ^[8]^和黑色素瘤^[9]^）的临床疗效有关。

过去几年中，PD-1抑制剂如pembrolizumab（帕博利珠单抗）和nivolumab（纳武利尤单抗）及PD-L1抑制剂atezolizumab（阿特珠单抗）已获批用于多种适应症，如黑色素瘤、NSCLC等。

## 3 现有临床数据及可能感染机制

关键的随机临床试验并没有显示接受PD-1或PD-L1抑制剂治疗的患者感染风险增加，但可出现irAEs ^[10-16]^。由阻断PD-1/PD-L1诱导的irAEs可能需要使用糖皮质激素、肿瘤坏死因子α (tumor necrosis factor α, TNF-α)拮抗剂（如英夫利昔单抗）、吗替麦考酚酯等药物进行暂时免疫抑制，可能继发感染。

一篇文章对接受PD-1/PD-L1抑制剂治疗的黑色素瘤患者进行了回顾性分析[其中52例仅接受纳武利尤单抗治疗，80例接受纳武利尤单抗和CTLA-4抑制剂伊匹木单抗(ipilimumab)联合治疗，83例接受帕博利珠单抗治疗]，发现有13例严重感染（累计发生率6.0%)，病原体以细菌为主，其中包括两例肺孢子菌肺炎（pneumocystis spneumonia, PCP)感染，亦有真菌、病毒感染。这些感染大多发生在接受过纳武利尤单抗和伊匹木单抗联合治疗的患者中。感染发生的主要危险因素是先前或同时接受糖皮质激素和/或TNF-α拮抗剂（英夫利昔单抗等）用于irAEs的治疗^[3]^。另一项针对167例接受纳武利尤单抗治疗的NSCLC患者的研究显示，共有33例患者(19.2%)出现33例感染，其中25例为细菌感染，2例为真菌，6例为病毒感染，糖尿病是感染发生的独立危险因素^[2]^。

值得注意的是，在除细胞毒化疗外没有irAEs或其附加免疫抑制治疗的情况下，近期仍有使用纳武利尤单抗或帕博利珠单抗治疗的患者出现潜伏结核感染重新激活的病例报道^[4, 17-19]^。这些患者潜伏结核的再活动十分迅速，在PD-1/PD-L1阻断开始后的前3个月内即出现。这些情况提示纳武利尤单抗或帕博利珠单抗的使用存在重新激活潜伏（休眠）结核感染的可能性，潜在机制可能为通过激活结核分枝杆菌特异性T细胞^[17]^，类似于艾滋病患者在开始接受抗逆转录病毒治疗时可能观察到的免疫重建炎症综合征(immune reconstitution inflammatory syndrome, IRIS)。根据上市后多中心注册记录，在法国（结核低患病率国家）接受PD-1/PD-L1抑制剂治疗的癌症患者中，活动性结核的发病率约为1/1, 000 ^[20]^。同时，2018年日本报道一例接受20个疗程纳武利尤单抗治疗后出现原有慢性进展性肺曲霉菌病(CPPA)急性加重的病例^[21]^。同年另一个使用纳武利尤单抗治疗的病例则被报道于治疗中出现水痘-带状疱疹病毒感染^[22]^。两例患者在治疗期间均未出现irAEs及使用免疫抑制治疗。提示PD-1/PD-L1抑制剂存在导致原有潜伏感染重新激活的潜在风险。

而另一方面，针对动物模型的体内研究确显示阻断PD-1/PD-L1引起的T细胞效应增强可能有利于提高免疫功能不全宿主或脓毒症免疫麻痹阶段中对某些感染，如细菌、真菌感染的免疫能力^[23-25]^。临床上有一例侵袭性毛霉菌病患者采用nivolumab和INF-γ治疗成功的报道^[26]^。对于PD-1/PD-L1抑制剂与感染的关系及潜在机制仍需进一步研究。

目前使用PD-1/PD-L1抑制剂继发感染的类型及特点如表 1所示。

**1:** PD-1/PD-L1抑制剂继发感染的类型及特点 Summary of PD-1/PD-L1 inhibitor related infections

Type	Possible mechanism	Risk factors	Common pathogens
Opportunistic infections related to irAEs	IrAEs required corticosteroids and/or immunosuppressants, leading to temporary immunesuppression	Use of corticosteroids and/or TNF-*α* inhibitors；Diabetes	Opportunistic infections caused by bacteria, fungi, virus, *et al*.
Reactivation of chronic/latent infections without irAEs	Resembling the IRIS; Boosting TH1 function^[17]^	Unknown	LTBI (10) CPPA (1) VZV (2)
irAEs: immune-related adverse event; IRIS: immune reconstitution inflammatory syndrome; LTBI: latent tuberculosis infection; CPPA: chronic progressive pulmonary aspergillosis; VZV: varicella zoster virus.			

## 4 诊断

### 4.1 治疗前监测

由于治疗期间可能需要免疫抑制治疗，开启PD-1/PD-L1抑制剂治疗前建议常规筛查潜伏/慢性感染。

#### 4.1.1 细菌感染

治疗期间应严密监测细菌感染，对于已出现严重感染或机会性感染的患者在开启PD-1/PD-L1抑制剂治疗前应审慎考虑。

#### 4.1.2 结核感染

对潜在需要PD-1/PD-L1抑制剂治疗的患者完善结核菌素试验、T-SPOT试验、γ干扰素释放试验（IGRA）及胸部X线或计算机断层扫描(computed tomography, CT)检查。对于存在可疑症状，影像见疑似病变，有活动性肺结核病史或不能进行影像学检查的患者，完善痰涂片及培养检查，必要时，可行支气管镜下灌洗或组织活检以明确诊断。

#### 4.1.3 HBV/HCV感染

尽管目前报道显示合并HBV/HCV感染的肿瘤病人使用PD-1/PD-L1抑制剂较非感染病人并不会出现药物毒性的增强^[27, 28]^，但考虑到发生irAEs应用生物制剂时可能导致HBV/HCV感染重新激活，以及PD-1/PD-L1抑制剂重新激活潜伏/慢性感染的潜在风险，建议在治疗前进行HBV/HCV感染的筛查。

#### 4.1.4 HIV感染

目前报道显示合并HIV感染的肿瘤病人使用PD-1/PD-L1抑制剂并不会显著增加风险^[29]^。但考虑到PD-1/PD-L1抑制剂重新激活潜伏/慢性感染的潜在风险，建议在治疗前进行HIV感染的筛查。

#### 4.1.5 其他

胸部CT检查可对慢性肺曲霉菌病进行检出。

### 4.2 治疗中及治疗后监测

治疗期间出现irAEs，需使用糖皮质激素或英夫利昔单抗进行免疫抑制治疗的患者，需进行密切监测，以早期发现感染迹象。高度建议包括肿瘤科及感染科医师参与的多学科诊治^[7]^。对于发生irAEs并使用抗TNF-α治疗（如英夫利昔单抗）的HBV/HCV携带者，在治疗期间及治疗后数月内均需要密切监测HBV/HCV病毒水平^[30]^。对于存在潜伏/慢性感染者，治疗期间需密切监测感染灶发展。

## 5 治疗

### 5.1 针对性治疗irAEs后继发感染

#### 5.1.1 抗感染治疗

肺炎是irAEs患者最易出现的感染类型^[2]^。对于出现感染征象的患者，建议立即开始经验性抗感染治疗。同时完善血清学及病原学检查，明确感染病原。PCP感染推荐使用复方磺胺甲恶唑，磺胺过敏的患者则可使用喷他脒等进行治疗^[30, 31]^。

#### 5.1.2 一般支持治疗

研究显示糖尿病为PD-1/PD-L1抑制剂继发感染的重要危险因素^[2]^。治疗时注意血糖控制。

#### 5.1.3 预防性治疗

(1) 使用强的松≥20 mg/d或等效的其他糖皮质激素至少4周的患者，建议用复方磺胺甲恶唑、阿托伐醌或喷他脒等预防肺孢子菌感染^[7, 31]^。(2)使用强的松≥20 mg/d或等效的其他糖皮质激素至少6周的患者，建议进行预防性抗真菌治疗（如氟康唑）^[30]^。(3)建议预防带状疱疹重新激活^[30]^。(4)不推荐预防性使用抗生素。现有研究显示抗生素的应用在使用免疫治疗的NSCLC患者中不利于预后^[32, 33]^。(5)疫苗：ESCMID指南建议依据临床常规进行疫苗接种^[7]^。但亦有学者^[31]^担心减毒活疫苗可能导致使用PD-1/PD-L1抑制剂治疗患者出现感染。对于灭活疫苗的研究结果则富有争议，一个小样本研究报道灭活疫苗增加irAEs发生风险，另一项研究则提示其对于使用PD-1/PD-L1抑制剂治疗患者是安全的^[34, 35]^。因此，在进行疫苗接种前，需谨慎评估其可能获益及风险。(6)与单独使用CTLA-4抑制剂伊匹木单抗或PD-1抑制剂（如纳武利尤单抗）相比，联合使用伊匹木单抗和PD-1抑制剂与更多irAEs显著相关^[9, 36, 37]^。

### 5.2 潜伏感染的重新激活

#### 5.2.1 一般治疗

注意血糖控制，密切监测感染灶变化。

#### 5.2.2 抗感染治疗

对于使用PD-1/PD-L1抑制剂期间出现潜伏结核重新激活的病人，建议进行抗结核治疗。考虑到PD-1/PD-L1抑制剂重新激活潜伏感染的报道较少，相关临床经验不足，抗结核药物的使用及疗程目前尚无定论。现有大部分病例在强化期治疗使用标准四联抗结核方案，亦有使用二联、三联及五联方案的报道^[4, 17]^。抗结核治疗期间，需密切监测肝功能，以及时发现肝损害情况，并与PD-1/PD-L1抑制剂相关药物毒性鉴别^[7, 31]^。

#### 5.2.3 是否需停用PD-1/PD-L1抑制剂？

目前对于使用PD-1/PD-L1抑制剂期间出现活动性肺结核的治疗仍缺乏足够临床资料。大体上，在出现活动性结核感染时，建议在抗结核治疗的同时暂时停用PD-1/PD-L1抑制剂，然而对于重新开始免疫治疗的时机目前尚未明确^[38]^。另外，亦有数例免疫治疗期间出现潜伏结核重新激活的病例在抗结核治疗的同时未停用PD-1/PD-L1抑制剂亦获得较好转归的病例报道^[4]^。

#### 5.2.4 预防性治疗

对于PD-1/PD-L1抑制剂使用前存在活动性肺结核的患者，建议进行抗结核治疗^[38]^。对于PD-1/PD-L1抑制剂使用前存在潜伏结核感染或可疑结核的患者，目前尚无预防性治疗的报道。

## References

[1] Brahmer JR, Govindan R, Anders RA (2018). The Society for Immunotherapy of Cancer consensus statement on immunotherapy for the treatment of non-small cell lung cancer (NSCLC). J Immunother Cancer.

[2] Fujita K, Kim YH, Kanai O (2019). Emerging concerns of infectious diseases in lung cancer patients receiving immune checkpoint inhibitor therapy. Respir Med.

[3] Del Castillo M, Romero FA, Argüello E (2016). The spectrum of serious infections among patients receiving immune checkpoint blockade for the treatment of melanoma. Clin Infect Dis.

[4] Picchi H, Mateus C, Chouaid C (2018). Infectious complications associated with the use of immune checkpoint inhibitors in oncology: reactivation of tuberculosis after anti PD-1 treatment. Clin Microbiol Infect.

[5] Nishimura H, Nose M, Hiai H (1999). Development of lupus-like autoimmune diseases by disruption of the PD-1 gene encoding an ITIM motif-carrying immunoreceptor. Immunity.

[6] Keir M, Butte M, Freeman G (2008). PD-1 and its ligands in tolerance and immunity. Annu Rev Immunol.

[7] Redelman-Sidi G, Michielin O, Cervera C (2018). ESCMID Study Group for Infections in Compromised Hosts (ESGICH) Consensus Document on the safety of targeted and biological therapies: an infectious diseases perspective (Immune checkpoint inhibitors, cell adhesion inhibitors, sphingosine-1-phosphate receptor modulators and proteasome inhibitors). Clin Microbiol Infect.

[8] Garon E, Rizvi N, Hui R (2015). Pembrolizumab for the treatment of non-small-cell lung cancer. N Engl J Med.

[9] Larkin J, Chiarion-Sileni V, Gonzalez R (2015). Combined nivolumab and ipilimumab or monotherapy in untreated melanoma. N Engl J Med.

[10] Balar A, Galsky M, Rosenberg J (2017). Atezolizumab as first-line treatment in cisplatin-ineligible patients with locally advanced and metastatic urothelial carcinoma: a single-arm, multicentre, phase 2 trial. Lancet.

[11] Robert C, Ribas A, Wolchok J (2014). Anti-eprogrammed-death-receptor-1 treatment with pembrolizumab in ipilimumab-refractory advanced melanoma: a randomised dose-comparison cohort of a phase 1 trial. Lancet.

[12] Powles T, Eder J, Fine G (2014). MPDL3280A (anti-PD-L1) treatment leads to clinical activity in metastatic bladder cancer. Nature.

[13] Topalian S, Hodi F, Brahmer J (2012). Safety, activity, and immune correlates of anti-PD-1 antibody in cancer. N Engl J Med.

[14] Brahmer J, Tykodi S, Chow L (2012). Safety and activity of anti-PD-L1 antibody in patients with advanced cancer. N Engl J Med.

[15] Eigentler T, Hassel J, Berking C (2016). Diagnosis, monitoring and management of immune-related adverse drug reactions of anti-PD-1 antibody therapy. Cancer Treat Rev.

[16] Fehrenbacher L, Spira A, Ballinger M (2016). Atezolizumab versus docetaxel for patients with previously treated non-small-cell lung cancer (POPLAR): a multicentre, open-label, phase 2 randomised controlled trial. Lancet.

[b17] 17Barber D L, Sakai S, Kudchadkar RR, *et al*. Tuberculosis following PD-1 blockade for cancer immunotherapy. Sci Transl Med, 2019, 11(475). pii: eaat2702. doi: 10.1126/scitranslmed.aat2702.

[18] Jensen KH, Persson G, Bondgaard A (2018). Development of pulmonary tuberculosis following treatment with anti-PD-1 for non-small cell lung cancer. Acta Oncologica.

[19] He W, Zhang X, Li W (2018). Activated pulmonary tuberculosis in a patient with melanoma during PD-1 inhibition: a case report. Onco Targets Ther.

[20] Picchi H, Mateus C, Chouaid C (2018). Infectious complications associated with the use of immune checkpoint inhibitors in oncology: reactivation of tuberculosis after anti PD-1 treatment. Clin Microbiol Infect.

[21] Uchida N, Fujita K, Nakatani K (2018). Acute progression of aspergillosis in a patient with lung cancer receiving nivolumab. Respirol Case Rep.

[22] Ursu R, Roumi A, Chouahnia K (2019). Varicella Zoster Virus vasculopathy in a patient treated with immune checkpoint inhibitor for lung cancer. Revue Neurologique.

[23] Brahmamdam P, Inoue S, Unsinger J (2010). Delayed administration of anti-PD-1 antibody reverses immune dysfunction and improves survival during sepsis. J Leukoc Biol.

[24] Lázár-Molnár E, Gácser A, Freeman G (2008). The PD-1/PD-L costimulatory pathway critically affects host resistance to the pathogenic fungus Histoplasma capsulatum. Proc Natl Acad Sci U S A.

[25] Patil N, Luan L, Bohannon J (2018). Frontline Science: Anti-PD-L1 protects against infection with common bacterial pathogens after burn injury. J Leukoc Biol.

[26] Grimaldi D, Pradier O, Hotchkiss R (2017). Nivolumab plus interferongamma in the treatment of intractable mucormycosis. Lancet Infect Dis.

[27] Kothapalli A, Khattak M (2018). Safety and efficacy of anti-PD-1 therapy for metastatic melanoma and non-small-cell lung cancer in patients with viral hepatitis: a case series. Melanoma Res.

[28] Tio M, Rai R, Ezeoke OM (2018). Anti-PD-1/PD-L1 immunotherapy in patients with solid organ transplant, HIV or hepatitis B/C infection. Eur J Cancer.

[29] Ostios-Garcia L, Faig J, Leonardi G (2018). Safety and efficacy of PD-1 Inhibitors among HIV-positive patients with non-small cell lung cancer. J Thorac Oncol.

[30] Thompson JA, Schneider BJ, Armand P (2019). Management of immunotherapy-related toxicities, version 1. 2019, NCCN Clinical Practice Guidelines in Oncology. J Natl Compr Canc Netw.

[b31] 31Cancer Care Ontario. Immune Checkpoint Inhibitor Toxicity Management.[06-11]. https: //www.cancercareontario.ca/en/content/immune-checkpoint-inhibitor-toxicity-management-clinical-practice-guideline.

[32] Mielgo-Rubio X, Chara L, Sotelo-Lezama M (2018). MA10.01 antibiotic use and PD-1 inhibitors: shorter survival in lung cancer, especially when given intravenously. type of infection also matters. J Thorac Oncol.

[33] Galli G, Poggi M, Fucà G (2018). MA10.02 impact of antibiotics on outcome of metastatic non small cell lung cancer patients treated with immunotherapy. J Thorac Oncol.

[34] Laubli H, Balmelli C, Kaufmann L (2018). Influenza vaccination of cancer patients during PD-1 blockade induces serological protection but may raise the risk for immune-related adverse events. J Immunother Cancer.

[35] Kanaloupitis DK, Chandran A, Ralph A (2017). Safety and efficacy of concurrent administration of influenza vaccine in patients undergoing anti-PD-1 immunotherapy. J Clin Oncol.

[36] Hodi F, Chesney J, Pavlick A (2016). Combined nivolumab and ipilimumab versus ipilimumab alone in patients with advanced melanoma: 2-year overall survival outcomes in a multicentre, randomised, controlled, phase 2 trial. Lancet Oncol.

[37] Postow M, Chesney J, Pavlick A (2015). Nivolumab and ipilimumab versus ipilimumab in untreated melanoma. N Engl J Med.

[38] Ho JC, Leung C (2018). Management of co-existent tuberculosis and lung cancer. Lung Cancer.

